# Enhanced crisis resilience of general practitioner-centred care: a retrospective cohort study of patients with coronary artery disease during the COVID-19 pandemic in Germany

**DOI:** 10.1186/s12875-025-02917-8

**Published:** 2025-07-14

**Authors:** Catriona Friedmacher, Dorothea Lemke, Renate Klaaßen-Mielke, Anastasiya Glushan, Angelina Müller, Kateryna Karimova

**Affiliations:** 1 Institute of General Practice, Goethe University Frankfurt, Theodor-Stern-Kai 7, Frankfurt, 60590 Germany; 2 Department of Medical Informatics, Biometry and Epidemiology, Ruhr University Bochum, Bochum, Germany

**Keywords:** Primary health care, Chronic disease, Coronary artery disease, COVID-19 pandemic, Continuity of patient care

## Abstract

**Background:**

Structured, comprehensive provision of primary care services has been shown to provide better outcomes in chronic disease management. In 2004, Germany introduced a programme of general practitioner (GP)-centred healthcare to strengthen the primary care sector. Crises such as pandemics, world conflict and climate events can result in significant challenges for the provision of routine healthcare requiring rapid reorganisation of existing models of care provision.

The objective of this study was to assess the impact of the COVID-19 pandemic on the provision of chronic disease surveillance services and the treatment of patients with coronary artery disease (CAD) by GPs in the federal state of Baden-Württemberg, Germany over the years 2019-2020 to examine if the previously demonstrated benefits of GPCC participation were maintained throughout the COVID-19 pandemic.

**Methods:**

Retrospective cohort study monitoring 170,466 CAD patients, conducted using biannually aggregated German insurance claims data (AOK-BaWü), comparing 2019 (pre-pandemic) with 2020 (COVID-19 pandemic), examining access (contacts), therapy (e.g. statin therapy), and clinical outcomes (acute myocardial infarction, angina pectoris, stroke, invasive procedures and pacemaker/defibrillator).

**Results:**

Patients enrolled in the GP-centred care programme (GPCC) had more frequent cohort-specific contacts, increasing during the pandemic, compared to those receiving standard care. Statin prescriptions were higher in the GPCC group and appear to be maintained over the study period. GPCC participation has demonstrated lower risks of all listed clinical outcomes in comparison to standard care and these established advantages of GPCC participation with respect to clinical outcomes were maintained during 2020 despite the challenges of the COVID-19 pandemic.

**Conclusion:**

Structured, comprehensive GP-centred care in Germany demonstrated resilience the challenges of the COVID-19 pandemic and was associated with better continuity of care for patients with coronary artery disease (CAD) and a maintained lower risk of CAD complications. These differences could be explained by the structured and comprehensive provision of primary care services and enhanced coordination with secondary care, allowing practices to maintain care effectively despite the challenges of the COVID-19 pandemic.

**Supplementary Information:**

The online version contains supplementary material available at 10.1186/s12875-025-02917-8.

## Introduction

The resilience of healthcare systems and routine delivery of care has in recent years been brought into focus due to global events such as pandemics, conflicts and climate changes [1]. For example, the outbreak of the COVID-19 pandemic in 2020 resulted in significant challenges for the healthcare sector worldwide. Mandatory lockdown restrictions and the cancellation or modification of routine services led to widespread disruption of chronic disease management and screening programmes. In a survey conducted by the World Health Organization in May 2020, 74% of countries in the European Region reported disruption to provision of outpatient services [1]. In the United States, a recent cross-sectional study demonstrated that in previously well-resourced areas with good primary care access before the COVID-19 pandemic, in-person ambulatory care appointments for chronic disease management dropped by 60–70%, accompanied by a 60–100% rise in telephone and video consultations [2]. Furthermore, a recent systematic review showed a median reduction of 37% of overall service provision in 20 countries examined, with highest reductions in outpatient care (42%) in comparison to admissions (28%), diagnostic procedures (31%) and therapeutic interventions (30%) [3]. In addition to changes in service provision, patients were also observed to have reduced their utilisation of ambulatory services, cancelling or postponing appointments due to fear of becoming infected [4]. In Germany, analysis from the Robert Koch-Institute demonstrated that during the first wave of the COVID-19 pandemic, nationwide general practitioner (GP) contacts reduced by 8.7% and outpatient specialist appointments by 12.3% [5]. Additionally, 35.5% of patients questioned in a nationwide survey had avoided or postponed medical care on at least one occasion, most frequently routine chronic disease monitoring appointments [6]. Maintaining preventative and curative services for non-communicable diseases, despite the challenges imposed on healthcare systems by major crises such as the COVID-19 pandemic, is critical for patients living with chronic diseases to prevent increased morbidity and mortality in this vulnerable group.

### GP-centred care programme in Germany

Patients with chronic diseases benefit from regular monitoring and follow-up to reduce the risk of disease-related complications [7–9]. Prevention, early recognition and regular chronic disease management are recognised to be core components of quality primary care [10–12]. Moreover, structured and comprehensive provision of primary care services has been shown to provide better outcomes in chronic disease management [13].

In Germany, fragmented care delivery [14] and lack of coordination between primary and secondary care has been a cause for concern. Routine GP care does not include a gatekeeping role, and there is no compulsory registration with a GP. This has hindered continuity of care and contributed to in higher costs [14]. In response to this, a law regarding the GP-centred care programme (GPCC) was introduced in Germany in 2004 and implemented in Baden-Württemberg (a large federal state in southern Germany with approximately 11.1 million inhabitants) by the Allgemeine Ortskrankenkasse (AOK) health insurance in 2008. The goal is to provide comprehensive, patient-centred care with a strengthened GP role as primary point of contact for patients and navigator through their healthcare journey, in comparison to an uncoordinated secondary care-led approach. The programme consists of a structured framework for the management of chronic diseases, continuous data-based quality improvement measures, the use of a computerised decision aid for prescribing medication and financial incentivisation mainly by capitation per enrolled patient and increased coordination between primary and secondary care. Enrolment is voluntary and enrolled patients are required to first consult with their GP, who can then facilitate secondary care specialist referral, where appropriate, in comparison to standard care where there is no formal GP gatekeeper role. The benefits of this approach have been clearly demonstrated, in particular a reduction in hospitalisation and 5-year mortality in both cardiovascular disease patients and high-risk patients, cost-effectiveness as well as a reduction in diabetes-related complications [12]. The overall uptake of GPCC in Baden-Württemberg has increased year on year, with approximately 1,78 million AOK insured individuals enrolled.

The aim of this study was to assess the impact of the COVID-19 pandemic on the provision of chronic disease surveillance services and the treatment of patients with coronary artery disease (CAD) by GPs in the federal state of Baden-Württemberg, Germany over the years 2019–2020 (i.e. the first year of the COVID-19 pandemic), comparing standard GP care with the structured, comprehensive GP-centred programme of care (GPCC) and to examine if the previously demonstrated benefits of GPCC participation were maintained throughout the COVID-19 pandemic.

## Methods

### Setting

The analysis was carried out as part of an evaluation GPCC in the German federal state of Baden-Württemberg (BaWü) and was fully approved by the ethics committee of Frankfurt University Hospital (Ref.No. 470/13). All participants in the study were insured by the largest regional statutory healthcare fund, AOK-BaWü, which has approximately 4 million members.

### Participants

To be eligible for inclusion in the study, participants were required to have coronary artery disease (CAD) (International Classification of Diseases, Tenth Revision, codes I20-I25), be current members of AOK-BaWü, reside in Baden-Württemberg, be 18 years of age or older, and not be enrolled in other healthcare programs. Patients in the GPCC group were required to have enrolled in the program prior to 2019, while patients in the standard care group needed to have an identifiable general practitioner. Individuals who switched to other healthcare providers during the observation period of 2019–2020 were excluded from the study. Deceased patients were treated as censored observations up to the time of death. The study design and inclusion criteria (i.e., available insurance data) ensured that there were no missing values. For each participant, characteristics of the enrolled practice are also collected: practice size (per 100 patients); urban-rural (categorical); group practice (yes/no). Patient and practice characteristics are described in Table 1.

**Table 1:** Patient and GP characteristics*

Covariates	Description
COVID-19 HZV	Interaction of COVID-19 year and type of care
HZV group	Type of care; reference HZV
COVID-19 - year 2020	Year; reference 2019
Half-year	1^st^ and 2^nd^; reference 1st half-year
Gender	Categorical; reference male
Care level	Care level > 0 (yes/no)
CCI score	General comorbidity score 0–18
Diabetes mellitus types 1 and 2 DMP participation	Yes/no
Age	In decades
Practice variables	Practice size (per 100 patients); urban-rural (categorical); group practice (yes/no)
Comorbidities	Heart failure, Diabetes mellitus (yes/no)
Diagnosis in previous 1 year	Atrial fibrillation, myocardial infarction, chronic kidney disease, hypertension, depression, dementia, malignancy, anxiety, obesity, nicotine use (yes/no)

*Abbreviations*: *GPCC *GP-centred care, *CCI *Charlson comorbidity index, *DMP D*isease management programme

*The characteristics are also the covariates of the analysis (ascertained at baseline)

### Study design and time frame of analysis

 We conducted a retrospective cohort-style monitoring study, based on administrative claims data provided by AOK-Ba-Wü, to examine how continuity of care evolved under GPCC versus usual-care conditions before and during the COVID-19 pandemic. All patients under GPCC and those receiving usual care were followed continuously over a two-year window (January 1, 2019 through December 31, 2020). Rather than predefining and selecting case or control events, we simply observed all care interactions and outcomes as they occurred, allowing us to capture real‐world variation without predisposition or outcome‐based sampling.

We structured our design around four equally spaced measurement points—every six months—so as to align half-year segments of the pandemic with their corresponding pre-pandemic halves (biannually for the years 2019 and 2020, with four measurement points: Control– 1^st^ and 2^nd^ half-year 2019; COVID-19 pandemic– 1^st^ and 2^nd^ half-year 2020). Each measurement interval represents a “wave” for comparative analysis, enabling us to contrast care continuity metrics (e.g., visit frequency, treatment gaps) in GPCC versus usual care across pandemic versus pre-pandemic periods.

### Primary outcomes

The primary outcomes of this analysis are displayed in Table 2. All outcomes are calculated annually (1st January − 31 December).

**Table 2:** Primary outcomes

Primary outcome indicator	ICD-10 codes*
Cohort-specific GP contact	I25.0, I25.1, I25.2, I25.5, I25.6, I25.8, I25.9
Statin prescriptions	ATC codes reported by AOK-BaWü
Myocardial infarction	I21.-
Angina pectoris	I20.-
Stroke	I61.-, I62.-, I63.-, I64.-
Pacemaker/defibrillator implantation	OPS codes reported by AOK-BaWü
Minimally invasive cardiac procedures	OPS codes reported by AOK-BaWü

*Reported outpatient or inpatient primary diagnosis code

### Statistical analysis

For the descriptive analysis of baseline characteristics, arithmetic means and standard deviations (SD) were calculated for continuous variables, while relative and absolute frequencies were reported for categorical variables.

The modelling of outcome indicators was conducted using Generalized Estimating Equations (GEE), which account for repeated measurements and intra-individual correlations over time. GEE is a highly suitable alternative for evaluating longitudinal data while appropriately accounting for correlated observations [15]. The choice of distribution and link function was based on the type of the dependent variable: for binary outcomes (i.e., occurrence of an event per half-year), a binomial distribution with a logit link function was assumed; for count outcomes (i.e., number of events per half-year), a negative binomial distribution with a log link function was applied. In the latter case, deaths were incorporated via an offset variable to reflect reduced observation times. Each outcome was modelled including the following fixed effects: (1) GPCC vs standard care (2) COVID-19-year effect (2020 vs. 2019 (3) Half-year effect (2^nd^*vs*. 1^st^ half-year) and (4) COVID-19 GPCC *vs*. standard care interaction effect. The models were additionally adjusted for several pre-specified covariates, which are listed in the Table 1 and are used as the covariates of exposure and outcome (Supplementary Table 1). To account for correlation due to repeated measures, a first-order autoregressive (AR1) correlation structure with homogeneous variances was specified.

Of particular interest was the interaction between type of care and COVID-19 year, which allowed us to assess whether patients receiving care in the GPCC model showed different outcome trends in 2020 compared to those in standard care. This provides insight into whether the GPCC model offered greater resilience in maintaining continuity of care during the pandemic.

To adjust for multiple testing across outcome indicators, Bonferroni correction was applied to both*p*-values and confidence intervals. While a significance level of 0.05 was retained for each outcome, Bonferroni adjustment resulted in 98.75% confidence intervals. The results are reported as odds ratios (OR) for binary outcomes and rate ratios (RR) for count outcomes, along with the respective *p*-values and confidence intervals (CI). For graphical illustration, the least-square means (LS Means) estimated from the model were shown half-yearly for the primary outcomes stratified for the type of care.

SAS version 9.4 (SAS Institute Inc., Cary, USA) was used for all statistical analysis.

## Results

At the start of the observation period in 2019 a total of 170,466 patients with CAD were identified in the AOK-BaWü population. 116,153 patients were enrolled in the GP-centred healthcare programme (GPCC) and 54,313 received standard GP care. Tables 3 and 4 show patient and practice characteristics at baseline. The mean age of the patients in both groups was 74.6±11.2 years and the distribution of gender in both groups was similar. However, there were several baseline differences between patients in GPCC and standard care groups. A higher proportion of patients in the standard care group had an assigned care level of 4 or 5 (higher score on 5-point scale equates to higher care requirements). Patients in the GPCC group had higher Charlson Comorbidity Index (CCI) scores consistent with a higher prevalence of chronic conditions and multimorbidity, in comparison to the standard care group. GP practices with high GPCC enrolment had larger number of AOK-BaWü patients, were more often located in rural areas and were more often group practices.

**Table 3:** Description of the baseline characteristics of the CAD cohort in 2019

	GPCC	Standard care	Total
	(*N*=116,153)	(*N*=54,313)	(*N*=170,466)
Age (mean±SD)	74.5±11.2	74.8±11.3	74.6±11.2
Gender			
Female	41.6% (48,273)	41.1% (22,305)	41.4% (70,578)
Male	58.4% (67,880)	58.9% (32,008)	58.6% (99,888)
CCI (mean±SD)	4.00 (2.70)	3.55 (2.68)	3.86 (2.70)
Care leve			
0	76.4% (88,755)	73.6% (39,949)	75.5% (128,704)
1	2.57% (2,982)	2.57% (1,394)	2.57% (4,376)
2	9.81% (11,398)	10.4% (5,674)	17,072% (10.0)
3	6.96% (8,087)	8.02% (4,358)	7.30% (12,445)
4	3.32% (3,861)	4.04% (2,193)	3.55% (6,054)
5	0.92% (1,070)	1.37% (745)	1.06% (1,815)
Comorbidity COPD	20.5% (23,769)	17.7% (9,593)	19.6% (33,362)
Comorbidity Asthma	9.08% (10,545)	7.67% (4,168)	8.63% (14,713)
Comorbidity Diabetes mellitus	40.8% (47,419)	38.8% (21,097)	40.2% (68,516)
Comorbidity Heart failure	40.3% (46,864)	28.5% (15,496)	36.6% (62,360)
Myocardial infarction in previous year	3.50% (4,061)	4.08% (2,217)	3.68% (6,278)
CKD in previous year	24.2% (41,183)	19.7% (10,694)	24.2% (41,183)
Hypertension in previous year	86.3% (100,235)	85.8% (46,607)	86.1% (146,842)
COPD in previous year	20.5% (23,769)	17.7% (9,593)	19.6% (33,362)
Depression in previous year	27.8% (32,281)	22.8% (12,389)	44,670% (26.2)
Dementia in previous year	6.31% (7,327)	8.57% (4,654)	7.03% (11,981)
Malignancy in previous year	17.8% (20,687)	17.2% (9,330)	17.6% (30,017)
Atrial fibrillation in previous year	23.6% (27,398)	23.1% (12,539)	23.4% (39,937)
Anxiety disorder in previous year	7.7% (8,939)	9.37% (5,088)	8.23% (14,027)
Obesity in previous year	25.2% (29,246)	24.9% (13,539)	25.1% (42,785)
Nicotine use in previous year	11.7% (13,641)	11.8% (6,407)	11.8% (20,048)
Enrolled in DMP-CAD	58.5% (67,919)	39.3% (21,372)	52.4% (89,291)
Enrolled in DMP-Asthma	2.73% (4,660)	1.72% (933)	2.73% (4,660)
Enrolled in DMP-COPD	7.95% (9,229)	4.40% (2,389)	6.82% (11,618)
Enrolled in DMP-Diabetes mellitus type 1 or 2	36.4% (42,231)	26.6% (14,434)	33.2% (56,665)

“In previous year” refers to new diagnosis in the preceding year

*Abbreviations*: *GPCC* GP-centred care, *CCI* Charlson Comorbidity Index, *COPD* Chronic obstructive pulmonary disease, *CKD* Chronic kidney disease, *DMP* Disease management programme, *CAD* Coronary artery disease, *SD* Standard deviation

**Table 4:** Description of practice characteristics in 2019

	GPCC	Standard care	Total
	(*N*=116,153)	(*N*=54,313)	(*N*=170,466)
Group practice	56.4%	43.0%	52.1%
Geographic designation			
Rural	51.0%	48.3%	50.1%
Urban	49.0%	51.7%	49.9%
Practice size (mean±SD)*	794.1±558.0	443.4±275.1	682.3±512.8

*Abbreviations*: *GPCC* GP-centred care, *SD* Standard deviation

*Practice size = average number of patients registered at a GP practice

**Fig. 1:**
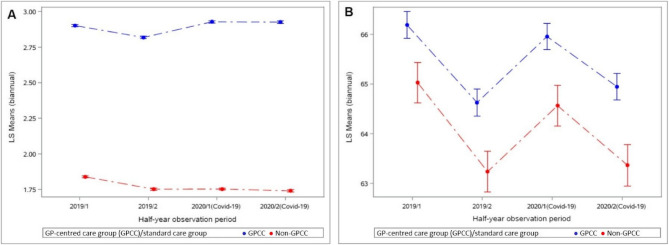
Estimated mean number of GP contacts per patient due to CAD diagnosis (**A**) and estimated proportion of patients with at least one statin prescription (**B**) per half-year-period in 2019 and 2020 (multivariable-adjusted model)

**Fig. 2:**
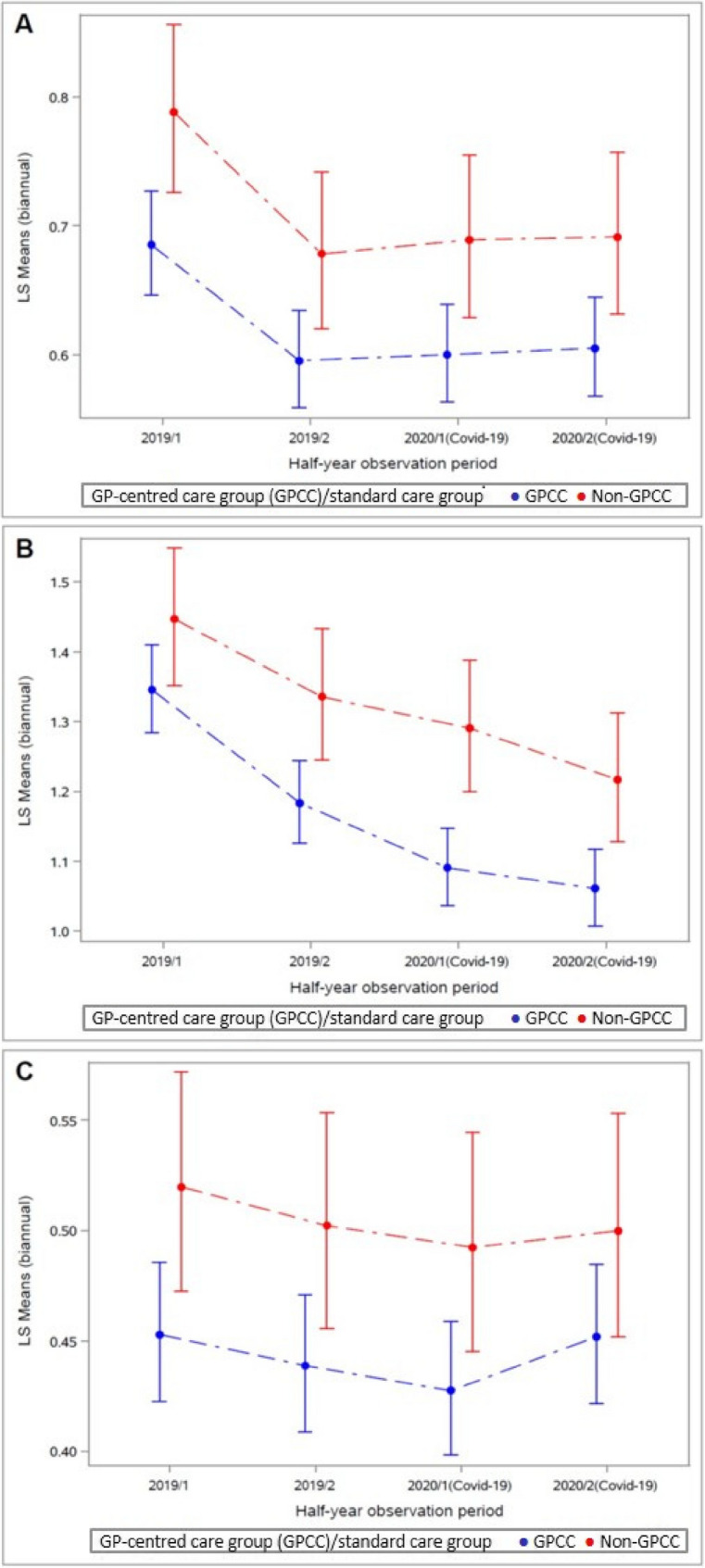
Estimated rate of myocardial infarction (**A**), angina pectoris (**B**) and stroke (**C**) per half-year-period in 2019 and 2020 (multivariable-adjusted model)

**Fig. 3:**
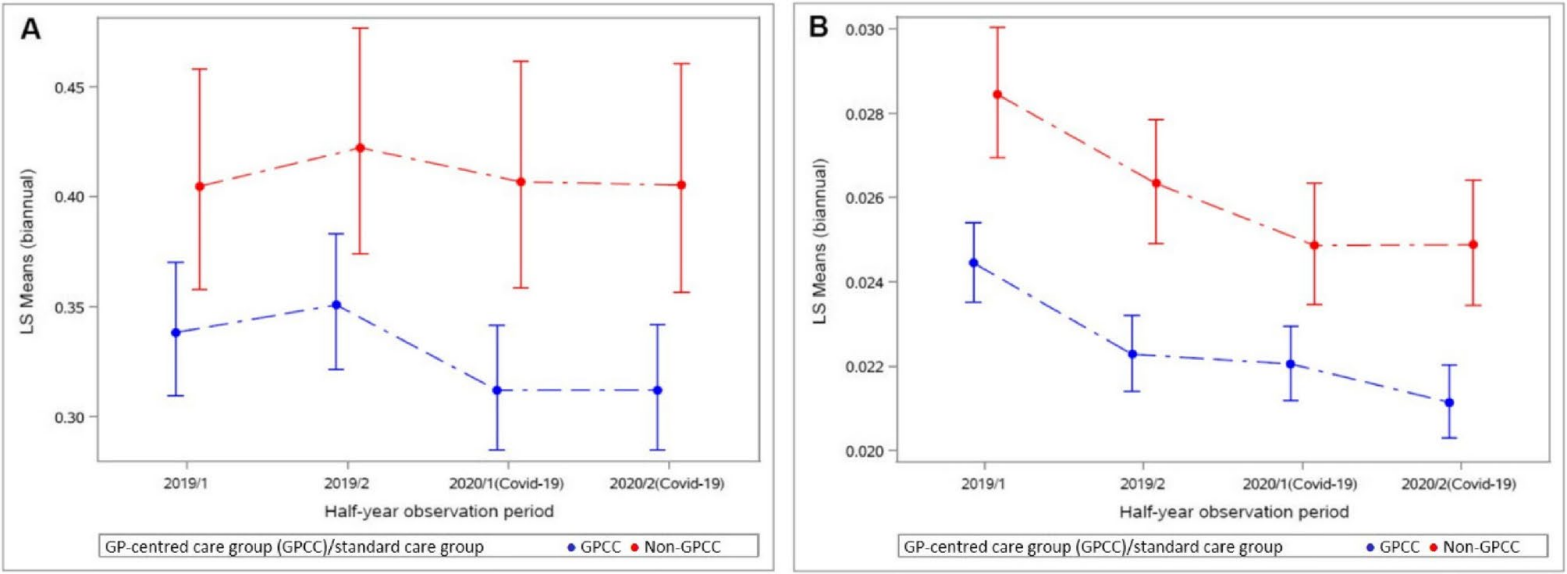
Estimated rate of pacemaker/defibrillator insertions (**A**) and estimated mean number of minimally invasive cardiac procedures (**B**) per half-year-period in 2019 and 2020 (multivariable-adjusted model)

In the multiple GEE model, participation in GPCC was shown to be significantly beneficial for all outcomes (Table 5). Patients enrolled in the GPCC had more frequent cohort(CAD)-specific GP contacts (Fig. 1A) in comparison with those receiving standard care (RR 1.63; 98.75% CI 1.62-1.64; *p*<0.001). COVID-19 year (2019 vs. 2020) alone did not have a significant impact on the frequency of GP contacts (RR 1.00; 98.75% CI 1.00-1.00; *p*=0.019). Over the study period 2019-2020, the interaction effect of GPCC group and COVID-19 pandemic showed an increase in cohort-specific GP contacts in the GPCC group (RR 1.05; 98.75% CI 1.05-1.06, *p*<0.001) in comparison to the standard care group. The probability of receiving one or more prescriptions for statin therapy (Fig. 1B) was higher in the GPCC group (OR 1.06; 98.75 % CI 1.04-1.08; *p*<0.001) but no COVID-19 effect was found. The interaction of COVID-19 year (2019/2020) and GPCC group showed no significant differences between GPCC and standard care in the continuity of statin therapy over the study period (OR 1.01; 98.75% CI 0.99-1.02; *p*=0.212). The risk of acute myocardial infarction (Fig. 2A) was lower in the GPCC group (OR 0.87; 98.75% CI 0.82-0.92; *p*<0.001) and there was no significant COVID-19-year effect (OR 0.94; 98.75% CI 0.89-1.00; *p*=0.034). Similarly, a lower risk was seen for angina pectoris (Fig. 2B) in the GPCC group (OR 0.88; 98.75% CI 0.84-0.93; *p*<0.001) with, in this case, a significant COVID-19-year effect (OR 0.88; 98.75% CI 0.84-0.91; *p*<0.001). Additionally, the risk of stroke (Fig. 2C) was lower in the GPCC group (OR 0.88; 98.75% CI 0.83-0.93; *p*<0.001) and there was no significant influence of COVID-19 pandemic year on new stroke diagnoses (OR 0.98; 98.75% CI 0.92-1.04; *p*=0.461). The risk of pacemaker/defibrillator insertions (Fig. 3A) was lower in the GPCC group in comparison to standard care (RR 0.80; 98.75% CI 0.74-0.86; *p*<0.001) and COVID-19 pandemic year (2019 *vs *2020) did not have a significant influence on new pacemaker/defibrillator procedures (OR 0.94; 98.75% CI 0.87-1.02; *p*=0.130). The risk of minimally invasive cardiac procedures (Fig. 3B) was lower in the GPCC group (RR 0.86; 98.75% CI 0.83-0.90; *p*<0.001) and COVID-19 year resulted in a significant reduction of procedures during the observation period (RR 0.92; 98.75% CI 0.89-0.95; *p*<0.001). The interaction effect of GPCC group and COVID-19 pandemic did not show any statistically significant effect for any of the measured clinical variables and the benefits of GPCC participation appear maintained.

**Table 5:** Regression model results

Effect Outcome	GPCC vs. standard care	COVID-19 effect (2020 vs. 2019)	Half-year effect (2^nd^nd vs. 1^st^ half-year)	COVID-19 GPCC vs. standard care interaction effect
GP contacts due CAD diagnosis	RR 1.63	RR 1.00	RR 0.98	RR 1.05
(98.75% CI)	(1.62–1.64)	(1.00–1.00)	(0.98–0.98)	(1.05–1.06)
	*p* < 0.001	*p* = 0.019	*p* < 0.001	*p* < 0.001
Statin therapy prescription	OR 1.06	OR 1.00	OR 0.94	OR 1.01
(98.75% CI)	(1.04–1.09)	(0.99–1.01)	(0.93–0.95)	(0.99–1.03)
	*p* < 0.001	*p* = 0.461	*p* < 0.001	*p* = 0.212
Myocardial infarction	OR 0.87	OR 0.94	OR 0.93	OR 1.00
(98.75% CI)	(0.81–0.94)	(0.88–1.01)	(0.87-1.00)	(0.87–1.15)
	*p* < 0.001	*p* = 0.034	*p* = 0.008	*p* = 0.989
Angina pectoris	OR 0.88	OR 0.88	OR 0.93	OR 0.95
(98.75% CI)	(0.83–0.94)	(0.83–0.92)	(0.88–0.98)	(0.85–1.05)
	*p* < 0.001	*p* < 0.001	*p* < 0.001	*p* = 0.194
Stroke	OR 0.88	OR 0.98	OR 1.00	OR 1.02
(98.75% CI)	(0.81–0.95)	(0.91–1.05)	(0.93–1.08)	(0.88–1.18)
	*p* < 0.001	*p* = 0.461	*p* = 0.967	*p* = 0.797
Pacemaker/defibrillator insertion	OR 0.80	OR 0.94	OR 1.02	OR 0.92
(98.75% CI)	(0.73–0.88)	(0.86–1.04)	(0.93–1.12)	(0.76–1.12)
	*p* < 0.001	*p* = 0.130	*p* = 0.626	*p* = 0.295
Minimally invasive cardiac procedures	RR 0.86	RR 0.92	RR 0.95	RR 1.02
(98.75% CI)	(0.82–0.91)	(0.88–0.96)	(0.91–0.99)	(0.94–1.11)
	*p* < 0.001	*p* < 0.001	*p* = 0.001	*p* = 0.597terva

*Abbreviations*: *GPCC *GP-centered care, *CAD *Coronary artery disease, *OR *Odds ratio, *RR *Rate ratio, *CI *Confidence interval

## Discussion

The results of this study demonstrate that despite the introduction of COVID-19 restrictions and the resulting re-structuring of primary care services, both continuity (consistency and regularity of GP contacts) and quality of care (lower risk of CAD complications in the GPCC group) were maintained in the GPCC group, demonstrating better crisis resilience in this care model. Cohort (CAD)-specific GP contacts increased in the GPCC group during the observation period, in contrast to both findings from the Robert Koch-Institute showing a nationwide reduction in GP contacts of 8.7% during the first wave of the COVID-19 pandemic [5] and international studies, demonstrating a widespread decrease in primary care contacts during 2020 [3, 16]. In Germany, at the start of the COVID-19 pandemic, general practices remained the first point of contact for both routine and COVID-19 patient care [17]. Furthermore, preventative care was maintained, as demonstrated by higher and constant statin therapy prescription rates in the GPCC group. This suggests that practices, which had a high uptake of this structured, comprehensive care model, were more resilient to the challenges imposed by COVID-19 intervention measures and were able to maintain effective delivery of chronic disease monitoring services [18].

All analysed primary outcome indicators were lower in the GPCC group and were not significantly affected by the onset of the COVID-19 pandemic, indicating that despite limitations imposed by COVID-19 restrictions, participation in the GPCC programme remained an effective primary care model for patients with CAD and is a significantly protective factor for CAD complications. This is consistent with previous studies demonstrating the benefits of this model of care for CAD patients and other chronic disease cohorts [8, 12, 19]. Overall declines in new diagnoses of myocardial infarction, angina pectoris and stroke during 2020 are consistent with both national and international observations during this time period [7, 20, 21]. Additionally, the marked reduction in invasive procedures is reflective of a pattern of Germany-wide deferrals of non-critical in-patient procedures, in order to maintain hospital capacity for the provision of COVID-19 patient care [22].

The findings that GP practices with high GPCC enrolment were larger, more often located in rural areas, and were more often group practices, may have had an impact on the capability of practices to adapt to the implemented COVID-19 measures. It may be that single-handed or smaller practices had greater challenges in reorganising their practices in comparison to larger group practices, where GPs are able to share and redistribute their workload [23]. Other studies have demonstrated that a lack of coordination with authorities and insecure funding were challenges for practices in adapting services during the COVID-19 pandemic, whereas a robust team structure and effective interdisciplinary communication seemed to be beneficial [21, 24, 25]. The secured funding structure and increased interprofessional coordination within the GPCC model may have aided in practice resilience.

International evidence has shown that the diversion of resources to COVID-19 patient care and reductions in routine care for non-communicable diseases had a negative impact on areas of chronic disease monitoring [23, 26–30] and preventative services and that reduced access to healthcare may have resulted in episodes of poorly controlled disease for patients with pre-existing chronic diseases [30]. A report by the Organisation for Economic Co-operation and Development examining provision of primary care services worldwide during the COVID-19 pandemic, described a reduction in routine primary care service provision ranging from an 11% reduction in Norway to a 51% reduction in the United States [31]. Germany as a whole saw a reduction of 39% in GP consultations during the first wave of the COVID-19 pandemic [31].

Recent research from the United States showed marked reduction in preventive screening and monitoring for cardiovascular diseases during the first wave of the COVID-19 pandemic, demonstrated by an 81-90% fall in testing for low-density lipoprotein cholesterol and a 52-60% fall in new initiation of statin therapy [10]. In addition to reductions in routine monitoring and in the initiation of therapy, delayed or missed diagnoses were observed for cardiovascular diseases such as acute coronary syndrome [32, 33], stroke [20, 34, 35], and hypertension [33]. Researchers examining excess mortality in the United States during March-April 2020 showed that about one third could not be attributed to COVID-19. Specifically, increases were noted in deaths due to cardiac disease and diabetes during this period [36]. These findings raised concerns regarding the timely diagnosis and continuing management of non-communicable diseases during the COVID-19 pandemic, and underline the need for strategies to ensure that quality of care is maintained and healthcare systems continue to function in the event of future crisis situations.

This study benefits from the large size of the population-based sample and the statistical modelling, which was used to analyse data. There was comprehensive access to inpatient, outpatient and prescribing data, with no missing values. There were, however, several potential limitations. Firstly, the claims data set did not contain clinical data such as disease severity grades and documentation of prescribed medication doses (i.e. days of supply). However, for the study design, administrative data was advantageous to assess hospitalisation rates and continuity of care. Secondly, participation in GPCC was not randomized, but voluntary. There is subsequently a natural selection bias, which was attenuated for by adjustment for multiple potential factors of influence. Our analysis relies on the assumption that the claims data (i.e. specifically coding quality, data availability and data transfer) are of high quality. In a clinical setting, there can be a risk of missed diagnoses, variation in coding patterns amongst professionals, and potential for coding inaccuracy.

This study demonstrates the resilience of the GP-centred care model in Baden-Württemberg, Germany when faced with crisis highlighted by the example of the COVID-19 pandemic. This structured, coordinated model of care with secure funding for practices, allowed for chronic disease care to be maintained despite challenges and should be considered for further expansion in Germany to ensure resilience of primary care provision in the event of further crises.

## Conclusion

The GP-centred care programme provided sustainable continuity of care for patients with CAD during the first year of the COVID-19 pandemic and was associated with a lower incidence of CAD complications. The previously demonstrated advantages of GPCC participation were maintained despite the challenges imposed by the COVID-19 pandemic, highlighting better resilience in crisis situations. These differences could be explained by the structured and comprehensive primary care model, including better coordination between primary and secondary care and secure funding for practices. 

## Supplementary Information

Supplementary Material 1.

## Data Availability

The data that support the findings of this study are available from the statutory health insurance provider AOK Baden-Württemberg but restrictions apply to the availability of these data, which were used under license for the current study, and so are not publicly available. Data codes used for analyses are however available from the authors upon reasonable request and with permission of AOK Baden-Württemberg. Requests should be addressed to co-author: Dr K. Karimova, Institute of General Practice, Goethe University, Theodor-Stern-Kai 7, 60590 Frankfurt, Germany.

## Abbreviations

GPCC: general practitioner centred care
CAD: coronary artery disease
BaWü: Baden Württemberg
CCI: Charlson comorbidity index
DMP: disease management programme
COPD: chronic obstructive pulmonary disease
CKD: chronic kidney disease
SD: standard deviation
OR: odds ratio
RR: rate ratio
CI: confidence interval
