# Supplementary material for: Enhanced crisis resilience of general practitioner-centred care: a retrospective cohort study of patients with coronary artery disease during the COVID-19 pandemic in Germany
Source: BMC Prim Care. 2025 Jul 14;26:225. doi: 10.1186/s12875-025-02917-8 (PMC12257840; doi:10.1186/s12875-025-02917-8)
Supplement: Supplementary file 1 — Supplementary Material 1. [file 12875_2025_2917_MOESM1_ESM.docx]

**Supplementary Table S1:** Overview of all GEE Models according to outcomes and covariates

|  | GP contacts due CAD diagnosis | | Statin therapy prescription | | Myocardial infarction | | Angina pectoris | | Minimally invasive cardiac procedures | |
| --- | --- | --- | --- | --- | --- | --- | --- | --- | --- | --- |
|  | RR  [97.75% CI] | p-value | RR  [97.75% CI] | p-value | OR  [97.75% CI] | p-value | OR  [97.75% CI] | p-value | RR  [97.75% CI] | p-value |
|  |  |  |  |  |  |  |  |  |  |  |
| COVID-19 GPCC vs. standard care interaction effect | 1.05 [1.05;1.06] | 0.000 | 1.01 [0.99;1.03] | 0.212 | 1.00 [0.87;1.15] | 0.989 | 0.95 [0.85;1.05] | 0.194 | 1.02 [0.94;1.11] | 0.597 |
| GPCC vs. standard care | 1.63 [1.62;1.64] | 0.000 | 1.06 [1.04;1.09] | 0.000 | 0.87 [0.81;0.94] | 0.000 | 0.88 [0.83;0.94] | 0.000 | 0.86 [0.82;0.91] | 0.000 |
| COVID-19 effect (2020 vs. 2019) | 1.00 [1.00;1.00] | 0.019 | 1.00 [0.99;1.01] | 0.461 | 0.94 [0.88;1.01] | 0.034 | 0.88 [0.83;0.92] | 0.000 | 0.92 [0.88;0.96] | 0.000 |
| Half-year effect (2nd vs. 1st half-year) | 0.98 [0.98;0.98] | 0.000 | 0.94 [0.93;0.95] | 0.000 | 0.93 [0.87;1] | 0.008 | 0.93 [0.88;0.98] | 0.000 | 0.95 [0.91;0.99] | 0.001 |
| Gender | 1.02 [1.02;1.03] | 0.000 | 1.54 [1.51;1.58] | 0.000 | 1.23 [1.15;1.32] | 0.000 | 1.17 [1.1;1.24] | 0.000 | 1.33 [1.26;1.39] | 0.000 |
| Age | 1.00 [1.00;1.00] | 0.889 | 0.9 [0.89;0.91] | 0.000 | 1.04 [1.00;1.08] | 0.009 | 0.88 [0.86;0.91] | 0.000 | 0.9 [0.88;0.92] | 0.000 |
| Charlson Comorbidity Index (CCI) | 1.02 [1.01;1.02] | 0.000 | 1.05 [1.05;1.06] | 0.000 | 1.41 [1.4;1.43] | 0.000 | 1.22 [1.21;1.24] | 0.000 | 1.36 [1.34;1.37] | 0.000 |
| Enrolled in DMP-Diabetes mellitus type 1 or 2 | 1.14 [1.13;1.15] | 0.000 | 1.47 [1.43;1.51] | 0.000 | 0.75 [0.69;0.82] | 0.000 | 0.87 [0.81;0.94] | 0.000 | 0.78 [0.74;0.83] | 0.000 |
| Practice size | 1.00 [1.00;1.00] | 0.000 | 1.00 [1.00;1.00] | 0.000 | 1.00 [1.00;1.00] | 0.158 | 1.00 [1.00;1.00] | 0.535 | 1.00 [1.00;1.00] | 0.049 |
| Geographic designation (urban/rural) | 0.98 [0.97;0.99] | 0.000 | 1.02 [1.00;1.04] | 0.051 | 1.08 [1.01;1.15] | 0.005 | 1.08 [1.02;1.14] | 0.001 | 1.00 [0.95;1.04] | 0.887 |
| Group practice | 1.03 [1.03;1.04] | 0.000 | 1.04 [1.02;1.06] | 0.000 | 1.02 [0.95;1.08] | 0.551 | 1.04 [0.99;1.1] | 0.061 | 1.00 [0.95;1.04] | 0.827 |
| Comorbidity Diabetes mellitus | 0.95 [0.95;0.96] | 0.000 | 0.97 [0.95;0.99] | 0.002 | 0.81 [0.74;0.88] | 0.000 | 0.81 [0.76;0.88] | 0.000 | 0.93 [0.88;0.98] | 0.001 |
| Atrial fibrillation in previous year | 0.96 [0.95;0.96] | 0.000 | 0.87 [0.85;0.89] | 0.000 | 0.82 [0.76;0.89] | 0.000 | 1.06 [0.99;1.13] | 0.04 | 1.06 [1.01;1.12] | 0.004 |
| Myocardial infarction in previous year | 1.00 [1.00;1.00] | 0.555 | 1.53 [1.47;1.59] | 0.000 | 3.53 [3.19;3.91] | 0.000 | 2.6 [2.37;2.85] | 0.000 | 1.96 [1.81;2.13] | 0.000 |
| CKD in previous year | 0.97 [0.97;0.98] | 0.000 | 1.00 [0.98;1.02] | 0.997 | 0.8 [0.74;0.86] | 0.000 | 0.89 [0.84;0.96] | 0.000 | 0.87 [0.83;0.92] | 0.000 |
| Hypertension in previous year | 1.01 [1.01;1.02] | 0.000 | 1.24 [1.21;1.27] | 0.000 | 1.06 [0.95;1.18] | 0.167 | 1.3 [1.19;1.42] | 0.000 | 1.31 [1.22;1.41] | 0.000 |
| Depression in previous year | 0.99 [0.99;1.00] | 0.029 | 0.85 [0.83;0.87] | 0.000 | 0.96 [0.89;1.03] | 0.159 | 1.09 [1.02;1.16] | 0.001 | 0.89 [0.84;0.93] | 0.000 |
| Dementia in previous year | 0.95 [0.94;0.96] | 0.000 | 0.75 [0.72;0.77] | 0.000 | 0.61 [0.53;0.7] | 0.000 | 0.61 [0.53;0.7] | 0.000 | 0.54 [0.48;0.6] | 0.000 |
| Malignancy in previous year | 0.95 [0.94;0.95] | 0.000 | 0.8 [0.78;0.83] | 0.000 | 0.41 [0.38;0.45] | 0.000 | 0.62 [0.57;0.67] | 0.000 | 0.52 [0.49;0.55] | 0.000 |
| Anxiety disorder in previous year | 1.01 [1.00;1.02] | 0.001 | 0.96 [0.93;0.99] | 0.001 | 0.92 [0.81;1.04] | 0.08 | 1.26 [1.15;1.39] | 0.000 | 0.97 [0.89;1.05] | 0.34 |
| Obesity in previous year | 1.02 [1.01;1.02] | 0.000 | 1.04 [1.02;1.06] | 0.000 | 0.87 [0.81;0.94] | 0.000 | 0.99 [0.93;1.06] | 0.838 | 0.88 [0.84;0.93] | 0.000 |
| Nicotine use in previous year | 1.01 [1.00;1.02] | 0.003 | 1.14 [1.11;1.18] | 0.000 | 0.97 [0.88;1.07] | 0.503 | 0.93 [0.86;1.01] | 0.027 | 1.16 [1.09;1.23] | 0.000 |

|  | Stroke | | Pacemaker/defibrillator insertion | |
| --- | --- | --- | --- | --- |
|  | OR [97.75% CI] | p-value | OR  [97.75% CI] | p-value |
|  |  |  |  |  |
| COVID-19 GPCC vs. standard care interaction effect | 1.02 [0.88;1.18] | 0.797 | 0.92 [0.76;1.12] | 0.295 |
| GPCC vs. standard care | 0.88 [0.81;0.95] | 0.000 | 0.8 [0.73;0.88] | 0.000 |
| COVID-19 effect (2020 vs. 2019) | 0.98 [0.91;1.05] | 0.461 | 0.94 [0.86;1.04] | 0.13 |
| Half-year effect (2nd vs. 1st half-year) | 1.00 [0.93;1.08] | 0.967 | 1.02 [0.93;1.12] | 0.626 |
| Gender | 0.93 [0.86;1.01] | 0.028 | 1.38 [1.24;1.52] | 0.000 |
| Age | 1.27 [1.22;1.33] | 0.000 | 1.09 [1.04;1.14] | 0.000 |
| Charlson Comorbidity Index (CCI) | 1.42 [1.41;1.44] | 0.000 | 1.18 [1.17;1.2] | 0.000 |
| Enrolled in DMP-Diabetes mellitus type 1 or 2 | 0.64 [0.58;0.7] | 0.000 | 0.87 [0.77;0.98] | 0.004 |
| Comorbidity Diabetes mellitus | 0.75 [0.69;0.83] | 0.000 | 0.92 [0.82;1.04] | 0.087 |
